# Complex Anatomy, Advanced Techniques: Microsurgical Clipping of a Ruptured Hypophyseal Artery Aneurysm

**DOI:** 10.3390/jcm14072361

**Published:** 2025-03-29

**Authors:** Corneliu Toader, Matei Serban, Nicolaie Dobrin, Razvan-Adrian Covache-Busuioc, Mugurel Petrinel Radoi, Alexandru Vlad Ciurea, Octavian Munteanu

**Affiliations:** 1 Department of Neurosurgery “Carol Davila”, University of Medicine and Pharmacy, 050474 Bucharest, Romania; corneliu.toader@umfcd.ro (C.T.); razvan-adrian.covache-busuioc0720@stud.umfcd.ro (R.-A.C.-B.); petrinel.radoi@umfcd.ro (M.P.R.); prof.avciurea@gmail.com (A.V.C.); 2 Department of Vascular Neurosurgery, National Institute of Neurology and Neurovascular Diseases, 077160 Bucharest, Romania; 3 Puls Med Association, 051885 Bucharest, Romania; 4 “Nicolae Oblu” Clinical Hospital, 700309 Iasi, Romania; 5 Neurosurgery Department, Sanador Clinical Hospital, 010991 Bucharest, Romania; 6 Medical Section, Romanian Academy, 010071 Bucharest, Romania; 7 Department of Anatomy, “Carol Davila” University of Medicine and Pharmacy, 050474 Bucharest, Romania; octavian.munteanu@umfcd.ro

**Keywords:** hypophyseal artery aneurysm, subarachnoid hemorrhage, microsurgical clipping, advanced neuroimaging, cerebrovascular surgery

## Abstract

**Background:** Ruptured intracranial aneurysms remain the subject of debate in their management, but the management of lesions located at high-risk locations, such as the hypophyseal artery, continue to prove to be a challenge in anatomical orientation and proximity to vascular structures. While endovascular therapies have changed the treatment paradigms, microsurgical clipping is the gold standard for wide-necked aneurysms for which endovascular techniques may be suboptimal. The successful treatment of a ruptured hypophyseal artery aneurysm in an elderly patient is described in this report, which highlights the importance of advanced imaging, careful technique, and new understanding of personalized aneurysm management. **Methods:** An 82-year-old woman was admitted with a thunderclap headache, alteration of consciousness and meningeal signs, suggestive of subarachnoid hemorrhage (SAH). A non-contrast computed tomography (CT) and digital subtraction angiography (DSA) confirmed a saccular 12 × 10 mm aneurysm with a broad 3.13 mm neck arising from the hypophyseal artery. The location and morphology of the aneurysm required microsurgical clipping, which was performed through a right pterional craniotomy. **Results:** Correct clip placement, complete exclusion of the aneurysm, and resorption of the subarachnoid blood were both observed on postoperative imaging. The neurological examination was completely normal, with no complications. Follow-up imaging at three months demonstrated stable, marked cerebral atrophy with compensatory ventricular enlargement without evidence of recurrence. **Conclusions:** This case illustrates the important role of micro-surgical clipping in anatomically complex aneurysms and its sustainable outcome and accuracy in cases where endovascular practices would have limitations. Advanced imaging, like three-dimensional DSA and intraoperative tools, have revolutionized precision surgery, allowing achievement of optimal outcomes, even for more-complicated cases. With an evolving, dynamic field and exciting new technologies coming to the fore—such as artificial intelligence to predict rupture risk and augmented reality navigation—decision-making and treatment of complex aneurysms will be optimized along secure pathways towards tailored, high-resolution treatment in the sense of personalized and yet high-precision care.

## 1. Introduction

Cerebral aneurysms, notably those leading to subarachnoid hemorrhage (SAH), continue to be one of the most dreaded diseases in neurosurgery and neurovascular medicine [1]. Ruptured aneurysms, which account for approximately 85% of nontraumatic SAH cases, have a poor prognosis, with mortality rates of up to 50% and considerable neurological sequelae among more than one-third of survivors [2,3]. The acute and life-threatening characteristics of aneurysmal SAH, which present with an excruciating headache and loss of consciousness followed by an immediate neurological deterioration, require urgent intervention [4,5]. Ruptured cerebral aneurysms continue to pose a challenge in terms of their unpredictable presentations, complex anatomy, and the risk of life-threatening complications, such as vasospasm, rebleeding, and hydrocephalus, even with decades of advances in both the domain of imaging and treatment modalities [6,7].

Microsurgical clipping, which was initially introduced by Walter Dandy in the 1930s, has retained its position as the gold-standard modality for the treatment of aneurysms, especially in cases that were not amenable to endovascular techniques [8,9]. Unlike coil embolization, clipping allows for definitive exclusion from circulation and has the advantage of proven long-term durability with recurrence rates of under 5% [10]. Yet, microsurgical clipping of aneurysms is highly dependent on careful preoperative planning, meticulous intraoperative technique, and intimate knowledge of the spatial anatomy of aneurysms, particularly in high-risk regions like the hypophyseal artery, where even the slightest miscalculation can end in disaster [11]. Recent advances in imaging technology have revolutionized the management of intracranial aneurysms.

Digital subtraction angiography (DSA), frequently with the addition of three-dimensional reconstructions, provides tremendous detail regarding aneurysm morphology and size, neck orientation, and relationship to important adjacent structures [12]. Additionally, intraoperative devices, including Doppler ultrasonography and indocyanine green (ICG) angiography, have increased surgical precision by providing intraoperative evaluation of vascular flow and facilitating the complete exclusion of the aneurysms while tolerating the bypass parent vessel [13]. These technologies are essential within anatomically challenging aneurysms in which the error margin is minuscule [14].

We report a case of a ruptured hypophyseal artery aneurysm, which is an anatomically complex and uncommon vascular lesion that was surgically managed. The patient was an elderly woman with significant comorbidities who presented acutely with SAH and ultimately underwent successful microsurgical clipping. This report illustrates the importance of early diagnosis, detailed imaging, and careful surgical treatment in complicated aneurysms, which positively impact outcomes. We hope to provide an additional perspective into the iterative nature of neurovascular surgical practice by focusing on some of the technical intricacies and surgical decision-making involved.

## 2. Case Presentation

An 82-year-old female patient arrived at our clinic with the sudden onset of an explosive thunderclap headache, which she described as devasting and consuming. This was coupled with crushing nausea and uncontrolled vomiting. The symptoms started several hours before admission and had not stopped. Given how abrupt and serious her presentation was, the patient came to medical attention. The patient was found to have an altered level of consciousness on admission. She was listless and needed repeated verbal and tactile stimulation to elicit responses. Her environmental engagement was inconsistent, and she was disoriented to time, but she remained oriented to person and place. Her neurological examination was documented as a Glasgow Coma Scale (GCS) score of 11, with eye-opening to verbal commands (E: 3), incoherent verbal response (V: 3), and purposeful localization to painful stimuli (M: 5). This score indicated moderate neurological compromise and merited an urgent and thorough work-up. The neurological examination was notable for pathological findings. 

The patient demonstrated significant nuchal rigidity, also known as resistance to passive flexion of the neck, classic signs of meningeal irritation. A comprehensive cranial nerve exam was performed. Pupils equally reactive to light, 3 mm in diameter, constricting briskly to 2 mm with direct light exposure bilaterally. Extraocular movements were full and smooth with no evidence of diplopia, nystagmus, or gaze palsy. Motor and sensory function of the face was intact, and there were no asymmetries during resting activities or voluntary facial movements (e.g., smiling and raising the eyebrows). Sensation remained intact in all branches of the trigeminal nerve (V1, V2, and V3), and her corneal reflexes were unimpaired. Swallowing and speech functions were like-wise preserved, with no dysarthria or dysphagia, ruling out lower cranial nerve dysfunction.

Motor Exam: Muscle tone was normal in all four extremities; no spasticity, rigidity or abnormal posturing noted. Joint range of motion was symmetrical and free from contraction and all major muscle groups scored at 5/5 on the Medical Research Council (MRC) scale of muscle strength. Deep tendon reflexes were symmetrical and brisk bilaterally, and there were no pathological reflexes elicited, including Babinski’s sign. Sensory examination demonstrated intact light touch, pinprick, and proprioception in the extremities. But tenderness to the posterior cervical region was present, giving rise to suspicion for meningeal irritation. Consciousness was monitored and maintained, with intact cerebellar function. The patient was able to quickly and accurately demonstrate finger-to-nose and heel-to-shin testing without dysmetria or intention tremor. No dysdiadochokinesia was seen on rapid alternating movements. Due to her lethargic state, balance and gait assessments were deferred for safety purposes.

**Figure 1:**
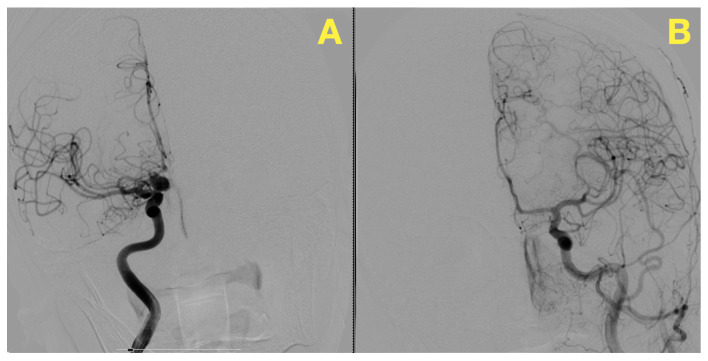
This lateral projection DSA image highlights a saccular aneurysm originating from the right hypophyseal artery. (**A**): The saccular aneurysm arising from the right hypophyseal artery is prominently visualized. The dome measures 12 × 10 mm, with a broad neck approximately 3.13 mm in diameter. This clear delineation of the aneurysm’s contour allows precise assessment of its morphology. (**B**): The adjacent branches of the internal carotid artery are unremarkable, with smooth contrast flow and no evidence of stenosis, collateralization, or irregularities, supporting the isolated nature of the aneurysmal pathology.

The constellation of symptoms—thunderclap headache, altered consciousness, and clear signs of meningeal irritation—were highly suggestive of a SAH. With urgency in the clinical setting and history of hypertension, urgent non-contrast computed tomography (CT) of the brain was performed to confirm the diagnosis and find the etiology of her presentation. The imaging showed hyperdensities in the sub-arachnoid spaces, primarily in the basal cisterns, and was suggestive of acute sub-arachnoid hemorrhage (SAH). This pattern was highly suggestive of the source of bleeding being an aneurysmal rupture. No intraparenchymal hemorrhage, hydrocephalus, or midline shift observed. The cerebroventricular system was structurally normal, and moderate cerebral atrophy, appropriate for the patient’s age, was exhibited. Bilateral carotid and vertebral angiography were performed to characterize the suspected aneurysm. An angiogram confirmed a right hypophyseal artery saccular aneurysm measuring 12 × 10 mm, with a neck diameter of 3.13 mm. The angioarchitecture, including the aneurysmal dome irregularity, was compatible with rupture. The study also revealed no significant abnormality of the other major intracranial vessels, confirming a normal vascular anatomy other than that previously mentioned. Preoperative DSA in lateral projection showed the saccular aneurysm originating from the right hypophyseal artery (Figure 1). The aneurysm was imaged with high contrast to the surrounding vascular structures, enabling detailed visualization of its morphology, as well as size and irregular shape confirmation. The characteristics observed previously: the absence of signs of vascular involvement of the surrounding vascular tree emphasized the isolated nature of the lesion.

**Figure 2:**
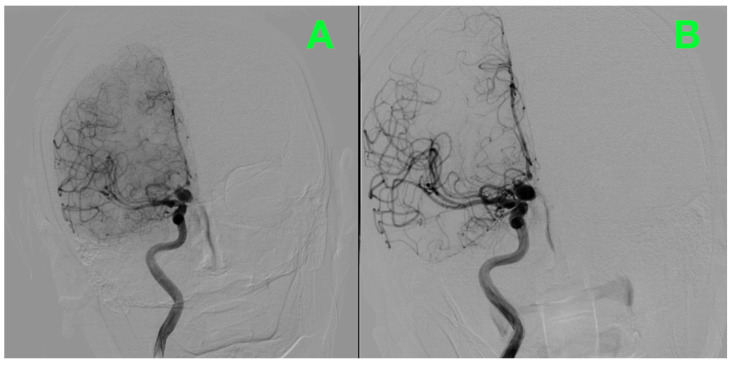
An oblique projection DSA image provides an alternative perspective of the right hypophyseal artery aneurysm. (**A**): The aneurysm’s spatial relationship to the hypophyseal segment of the internal carotid artery is evident. This view highlights the broad-based neck’s orientation, a critical factor in determining the approach for microsurgical clipping. (**B**): Adjacent arterial branches are shown with uninterrupted contrast flow, confirming the absence of any additional vascular abnormalities, stenoses, or arterial wall irregularities.

Figure 2 presents an alternative projection of the DSA, arising from a separate viewpoint, illustrating anatomical relationships with neighboring arteries, most interestingly, its location to the internal carotid artery. This projection demonstrates the neck of the aneurysm, which was roughly 3.13 mm in diameter, and was cruelly considered when deciding if microsurgical clipping would be feasible. The vast saccular dome is also demonstrable, which highlights the considerable hemodynamic stresses at this location, aligning with the angiographic evidence of rupture.

**Figure 3:**
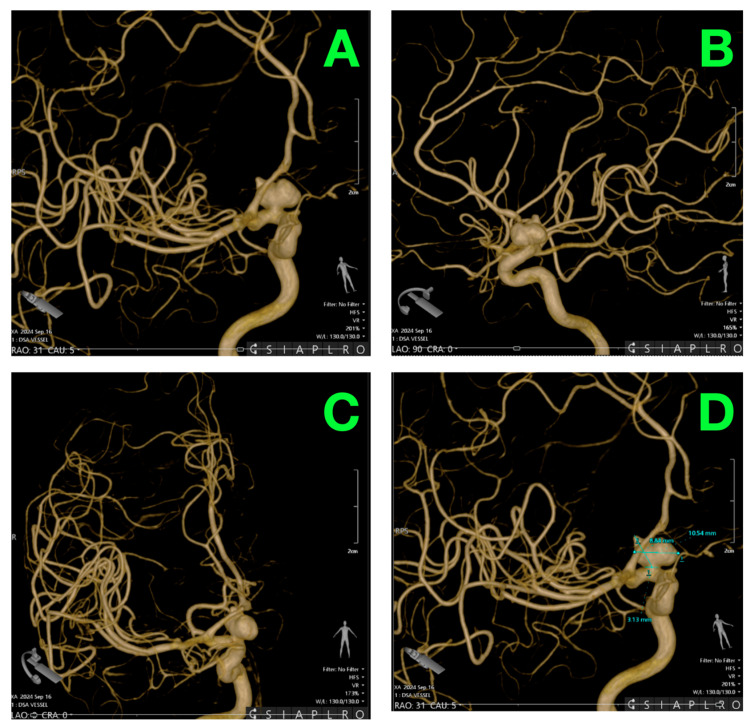
3D Reconstruction of cerebral vasculature. (**A**): A three-dimensional reconstruction of the cerebral vasculature demonstrates the saccular aneurysm originating from the right hypophyseal artery. The aneurysm’s broad neck and dome are prominently visualized, showing a well-defined connection to the parent vessel. This view emphasizes the aneurysm’s spatial relationship to the internal carotid artery, aiding in preoperative localization and surgical planning. (**B**): This projection offers an alternative angle, providing greater detail of the aneurysm’s orientation in relation to the intracranial arterial tree. The relationship between the aneurysm and surrounding vascular structures, including the anterior and middle cerebral arteries, is clearly displayed, illustrating the absence of additional vascular irregularities. (**C**): A more lateral view of the aneurysm highlights its attachment to the hypophyseal segment of the internal carotid artery. The contour and dimensions of the aneurysm are shown in high resolution, emphasizing its saccular configuration. The adjacent vascular branches are displayed in their normal anatomical alignment, further supporting the isolated nature of the lesion. (**D**): This detailed view incorporates precise measurements of the aneurysm. The dome measures 10.54 mm in height and 12 mm in width, with a neck diameter of 3.13 mm. These measurements are critical for surgical planning, particularly for determining the optimal clip size and placement during aneurysm clipping. The spatial reconstruction also provides a volumetric perspective of the aneurysm’s interaction with the parent artery, offering invaluable insight into its morphology and surgical accessibility.

Figure 3 depicts a detailed three-dimensional reconstruction of the cerebral vasculature, which can give a better spatial understanding of the aneurysm. The stereoscopic image illustrates not only the orientation of the aneurysm along with surrounding vascular structures but also its origin off the hypophyseal segment of the internal carotid artery. Reconstruction from that is especially helpful for the planning of the procedure, ensuring good localization of the lesion and the best strategy for placement of the clips, whilst sparing other vascular structures nearby.

The imaging together provided strong evidence to suggest a ruptured right hypophyseal artery aneurysm which required surgical intervention in an extremely urgent manner. The patient was taken to the operating room, where she was brought under general anesthesia, and her head was stabilized in a Mayfield head clamp. Again, the head was gradually turned toward the left and raised forward a little to obtain the surgical approach according to the right pterional corridor with direct access to basal cisterns and the hypophyseal region. A right-sided pterional craniotomy was performed to minimize the amount of brain retraction and maximize exposure. The scalp incision was curvilinear and extended from just anterior to the tragus and curved superiorly to the hairline. The temporalis muscle was also dissected carefully and elevated with retraction, and the bone flap was removed. Its dura mater was taut but intact, with the incision made semi-circularly along its fibers and reflected to expose the frontal and temporal lobes.

Microsurgical dissection was initiated with splitting of the sylvian fissure, which was meticulously split under high magnification with fine-tipped instruments. This step demanded careful artistry to maintain the fragile arachnoid layers, as well as the underlying neurovascular structures. To achieve brain relaxation, we aspirated cerebrospinal fluid from the basal cisterns, which improved visualization and reduced the amount of retraction necessary. The aneurysm was discovered to be a saccular outpouching that arose from the hypophyseal segment of the right internal carotid artery. The intraoperatively measured size was consistent with the preoperative angiographic findings: broad neck of 3.13 mm with dome size of 12 × 10 mm. Parts of the dome were uneven, with signs of thinning indicating rupture. Its proximity to important vascular structures, such as the optic nerve and anterior cerebral artery, necessitated meticulous dissection to prevent collateral damage. Temporary aneurysm clips were placed just proximal and distal to the aneurysm on the internal carotid artery to arrest blood flow and obtain a bloodless field.

The aneurysmal sac was dissected free from surrounding adhesions, allowing controlled manipulation of the sac. Temporary clips were applied in a timed and meticulous manner in order to minimize persistent ischemia to downstream territories. With high-powered magnification and gradual, precise maneuvers, a titanium permanent microsurgical clip was then applied across the aneurysmal neck. The position was precisely modified to exclude completely the aneurysm from circulation and maintain the patency of the parent artery.

**Figure 4:**
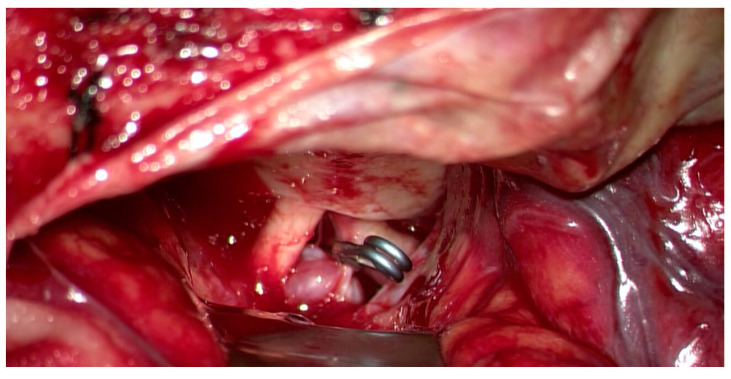
Intraoperative photograph. Intraoperative view of the right hypophyseal artery aneurysm following successful clipping. The titanium clip is precisely placed across the aneurysmal neck, ensuring complete exclusion of the aneurysm from the circulation while preserving the patency of the parent vessel. The meticulous dissection of the surrounding arachnoid layers and vascular structures is evident, highlighting the surgeon’s precision in isolating the aneurysm without compromising adjacent arteries.

Figure 4 also provides an intraoperative view of the aneurysm clip and adjacent structures that led to precise closure of the clipping process. The clip position was then carefully examined under the surgical microscope to ensure accurate placement and efficient clipping of the aneurysmal neck.

Hemostasis was achieved using gentle bipolar coagulation, with particular care to avoid thermal injury to adjacent structures. The operative field was irrigated thoroughly with warm saline to clear residual blood and debris, ensuring optimal visualization during closure. The dura mater was closed in a watertight fashion using interrupted sutures, and the bone flap was replaced and secured.

**Figure 5:**
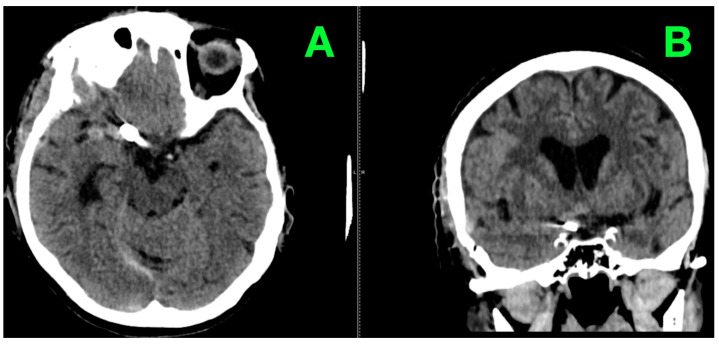
Postoperative non-contrast CT. (**A**): presents an axial view where the metallic artifact of the clip is clearly visible, verifying its proper positioning relative to the internal carotid artery. Surrounding structures demonstrated no evidence of residual or new hemorrhage, and there were visible signs of subarachnoid blood resorption within the basal cisterns. (**B**): a coronal reconstruction provides additional confirmation of the clip’s alignment at the hypophyseal segment of the internal carotid artery. The cerebroventricular system remained normal, with no evidence of midline shift, hydrocephalus, or other abnormalities. The basal cisterns appeared clearer, reflecting ongoing resolution of the subarachnoid hemorrhage.

The scalp incision was closed in anatomic layers with absorbable sutures for the deeper tissues and staples for the skin, providing a strong and cosmetic result. The procedure was well tolerated, and the patient was transferred to the intensive care unit for close monitoring. Postoperative non-contrast CT was performed to assess the result of surgical intervention (Figure 5). Imaging confirmed that the aneurysm clip was correctly placed at the right carotid cistern level.

**Figure 6:**
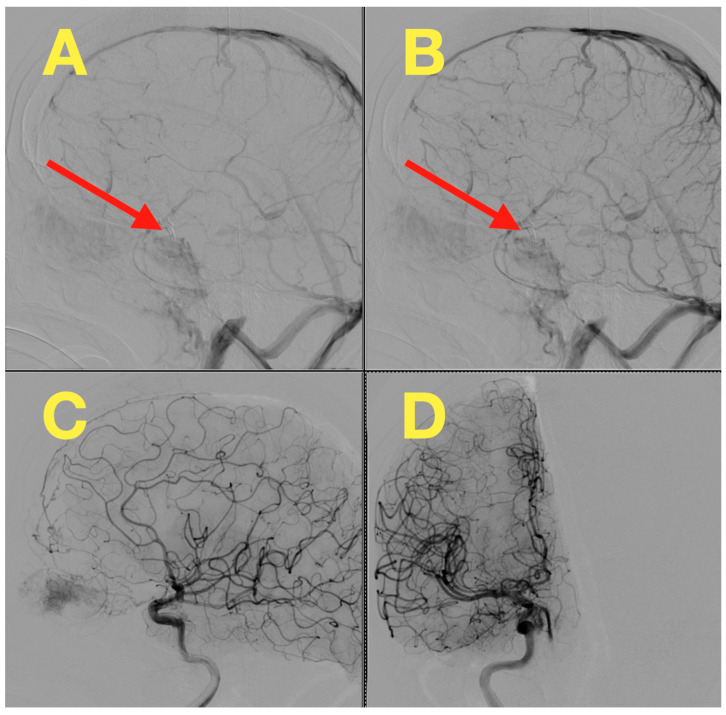
Postoperative DSA. (**A**): Lateral projection illustrating complete obliteration of the aneurysmal sac (indicated by red arrow), with no evidence of residual contrast filling or aneurysmal remnant. (**B**): Another lateral projection confirms stable exclusion of the aneurysm and preservation of the parent vessel, with smooth contrast flow through the internal carotid artery. (**C**): Oblique view demonstrates normal perfusion of the distal cerebral vasculature, with no signs of vasospasm, stenosis, or delayed contrast transit. (**D**): Contralateral projection highlights the integrity of the intracranial arterial circulation, further confirming the absence of vascular irregularities or collateral flow patterns.

To further confirm aneurysmal exclusion and vessel patency, a postoperative DSA was also performed. The study demonstrated complete obliteration of the aneurysm without residual filling, as well as preserved flow through the internal carotid artery and its branches. No complications such as vasospasm, stenosis, or delayed perfusion were observed, reinforcing the effectiveness of the microsurgical clipping (Figure 6).

**Figure 7:**
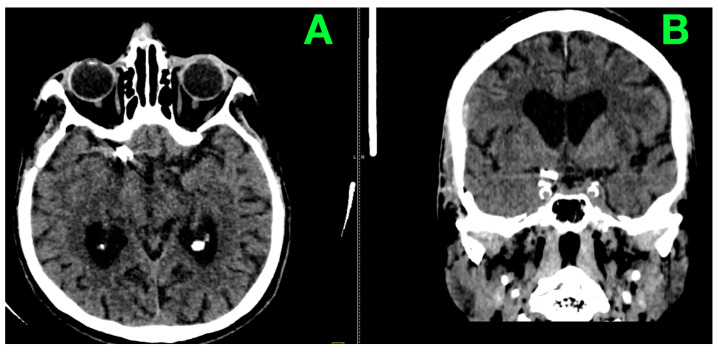
Three-month postoperative non-contrast CT scan. (**A**): Axial CT scan demonstrates the presence of the right temporofrontal craniotomy flap, with no evidence of complications at the surgical site. The aneurysm clip remains in its correct position at the right carotid cistern, with no associated artifact-related distortions affecting adjacent structures. The basal cisterns and cerebrospinal fluid spaces are clear, and there is no evidence of active or recent hemorrhage. (**B**): Coronal CT reconstruction highlights marked cerebral atrophy, with compensatory dilatation of the lateral ventricles. The ventricular system demonstrates no obstruction, and the midline structures remain symmetrically aligned. The absence of hemorrhagic changes or ischemic complications confirms the stable postoperative outcome.

A discharge plan detailed follow-up as well as rehabilitation direction designed to maximize her recovery. No neurological complaints were recorded during the 3 months postoperatively; functional independence was excellent; and no recurrence of symptoms was observed. The neurological examination of the patient was completely normal at the three-month control. On examination, she was alert, cooperative and orientated with a GCS of 15. No neurological deficits were observed, and there were no signs of meningeal irritation or sphincter control disturbances. An unenhanced CT performed at this time (Figure 7) confirmed the stability of the surgical result.

This case illustrates the successful microsurgical management of a ruptured right hypophyseal artery aneurysm. Timely diagnosis, precise surgical clipping, and comprehensive postoperative care resulted in complete aneurysmal exclusion and full neurological recovery, emphasizing the importance of meticulous intervention in complex neurovascular cases.

## 3. Discussion

Ruptured cerebral aneurysms, especially in more anatomically complex sites, such as the hypophyseal artery, are a marriage of technically astute physicians, technological advancements in imaging, and patient-specific treatment planning. This paper aims to bridge available knowledge with lessons learnt, as per our aforementioned case, to address complexities of treatment selection, long-term expectations and next steps. Intracranial aneurysms are heterogeneous in their morphology and pathophysiology, which require different treatment strategies. Saccular (berry) aneurysms, the most conservative form, develop at vascular bifurcation points as a result of chronic hemodynamic stress and are easily amenable to both microsurgical clipping and endovascular coiling [15]. Fusiform aneurysms with diffuse arterial dilatation without a discrete neck are less amenable to treatment with traditional devices and are often treated with flow-diverting stents or bypass procedures given their complex structure. Another form of dissection aneurysm, common in the vertebrobasilar system, arises from rupture of the vessel wall, with high risk of ischemia and rupture, leading to the potential need for endovascular stenting and/or vessel sacrifice with bypass reconstruction [16].

Mycotic aneurysms from infectious emboli have inflamed and friable walls and are treated primarily with antimicrobials, whereas management of progressive or ruptured disease is by surgical or endovascular means [17]. Familiarity with these schemas aids in choosing the best treatment modality, as aneurysm morphology has a significant impact on the treatment modality (microsurgical versus endovascular). Microsurgical and endovascular approaches continue to be the subject of ongoing debate in neurovascular surgery. Microsurgical clipping provides certain exclusion of the aneurysm, especially for complicated lesions such as wide-necked aneurysms or those in surgically accessible territories [18,19]. Clipping is known to have demonstrated durability, with a recurrence rate of ≤5%, especially at longer follow-up times, and is suggested as the best treatment for patients with anatomically challenging cases [20]. Minimally invasive endovascular approaches, such as coil embolization, stent-assisted coiling, and flow diverters, have gained popularity with a lower immediate procedural risk [21]. They also demonstrated that, at one year, coiling compared to clipping has lower morbidity and mortality, especially if applied to small uncomplicated anterior circulation aneurysms, as shown by the ISAT (International Subarachnoid Aneurysm Trial) [22].

However, the treatment failure rate of aneurysms treated with coils is between 20 and 33% in systematic reviews, frequently requiring re-treatment [23]. In this case, the ruptured saccular morphology of the aneurysm made the microsurgical clipping the favorable option. Moreover, wide-necked aneurysms that necessitate the use of endovascular approaches (like flow diverters) require long-term antiplatelet treatment, which has a high complication and morbidity rate in older patients or patients with comorbid risk factors, such as hypertension. No clip placement-related mortality was noted, corroborating with findings by Kranawetter et al., which proposed that clipping remains the treatment of choice for wide-necked or morphologically complex aneurysms [24]. Direct visualization and manipulation of the aneurysm intra-operatively allows for precision in ensuring comprehensive exclusion and parent artery preservation [25]. Table 1 below distils key publications and their relevance to this case, with emphasis on treatment mechanisms, outcomes, and technological innovations.

The development of in vivo imaging technology has revolutionized the management of intracranial aneurysms. The preoperative imaging in this case consisted of DSA with 3D reconstructions, allowing detailed assessment of both the aneurysm’s morphology and neck width, as well as spatial relationships with critical structures, including the optic nerve and internal carotid artery. These insights guided the surgical planning and execution, which were performed meticulously. Novel imaging modalities, such as high-resolution vessel wall imaging (HR-VWI), may enable new avenues of investigation in aneurysm research. HR-VWI is able to identify inflammatory changes and structural impairment in the aneurysmal wall and offer a new modality for rupture risk stratification [34,35]. Although not used in this instance, its increasing use in clinical practice suggests that it will soon be routine in risk stratification. Surgical treatment of hypophyseal artery aneurysms has its own challenges because they are adjacent to important neurovascular structures, such as the optic apparatus, pituitary gland, and internal carotid artery [36]. This approach affords optimal access while minimizing brain retraction, and, in this case, a pterional craniotomy was utilized. As has been universally recommended in the literature, the temporary clipping of the parent artery as performed here minimizes the risk of intraoperative rupture.

The landmark paper of Yasargil highlighted that meticulous use of this technique scrutinizes the aneurysmal dome, allowing systematic segmentation, resulting in surgical control over the aneurysmal dome and secure clipping [37]. High-powered microscopy dissection was crucial to free the aneurysmal neck without injury to surrounding structures. These steps are in line with findings from multiple large surgical series demonstrating direct clipping as the optimal method of obtaining a durable reconstruction of wide-necked aneurysms [38]. This case demonstrates the role of patient-specific factors (i.e., advanced age and hypertension) in treatment planning. Older patients tend to carry a higher surgical risk, but older age also reduces the applicability of endovascular strategies necessitating prolonged antiplatelet therapy, such as flow diverters. Studies by Stroh-Holly et al. suggest that in high-volume centers, microsurgical clipping may have similar morbidity rates as the endovascular route, even amongst geriatric patients [39]. The patient’s excellent neurologic recovery and imaging at three-month follow-up highlight the durability of microsurgical clipping. Long-term data demonstrating the persistence of clipping’s efficacy for wide-necked lesions supports the absence of residual aneurysm, as well as rebleeding and ischemic complications. Aneurysms require long-term monitoring. Although the American Heart Association/American Stroke Association guidelines recommend routine imaging follow-up to assess continued durability, recurrence is rare after clipping. Noninvasive imaging techniques such as CTA or MRA are now widely used in routine long-term follow-up because of their accuracy and safety [40,41]. This also illustrates disparities in access to advanced neurovascular care. However, access to advanced imaging modalities or experienced microsurgeons is limited in many low-resource settings. Filling these gaps is important in order to ensure equitable outcomes in aneurysm management. We can safely say the future of aneurysm management will be defined by the adoption of AI and machine learning. Machine learning predictive models trained on large datasets are under development in an attempt to quantify rupture risk and inform treatment planning [42,43].

Moreover, novel endovascular technologies, such as bioresorbable stents and intrasaccular flow disruptors, could offer minimally invasive treatments for complex aneurysms [44]. Despite such advancement, microsurgical clipping will still be necessary for anatomically complex lesions, especially those that need direct visualization and manipulation. Ongoing advancements in surgical techniques, such as robotic assistance and augmented reality navigation, will continue to enhance the contributions of clipping to the neurovascular armamentarium [45]. However, although spontaneous aneurysms are the most common cause of aneurysmal SAH, TIAs are a separate and important clinical entity. TIAs are most commonly secondary to blunt or penetrating head trauma, vascular injury, or iatrogenic manipulation. In contrast to spontaneous aneurysms, which develop as a result of untreated chronic hemodynamic stress, TIAs usually present with irregular shapes, tenuous walls, and an increased incidence of delayed rupture days or weeks after the initial insult [46]. The unpredictable nature of TIAs with respect to their course and anatomy makes their management uniquely challenging. Although small, stable TIAs can be watched with serial imaging, many do need to be addressed quickly to avert disastrous rebleeding. Endovascular options such as coil embolization and stent-assisted approaches are used frequently, especially for deep or surgically challenging aneurysms. In cases of significantly compromised vessel integrity, microsurgical interventions can include direct repair or bypass grafting [47]. The surgical treatment of hypophyseal artery aneurysms has remained one of the most technically difficult problems in cerebrovascular surgery. The complex anatomy, proximity to the optic apparatus and pituitary structures, and the frequent association with broad-neck configurations of such lesions demand detailed surgical planning and execution. Despite the evolution of endovascular techniques, such as coiling and flow diversion, the treatment of hypophyseal artery aneurysms with these modalities is limited by high rates of recurrence and the requirement for long-term antiplatelet therapy in flow-diverter cases. On the other hand, microsurgical clipping offers definitive exclusion of the aneurysm with immediate structural reinforcement around the skull base, which is especially beneficial for those with potential instability and those patients in whom endovascular treatment is inadequate [48].

Our case corroborates the findings in large surgical series, where microsurgical clipping provided for superior long-term stability and rates of aneurysmal obliteration for broad-necked hypophyseal artery aneurysms. Intraoperative direct manipulation of the aneurysm, precise clip placement, and the ability to attain complete exclusion make this technique remain relevant. Such consistency of outcomes in large cohorts confirms the generalizability of the principle of patient-centered treatment-choice dynamics as it can be applied to the surgical treatment of hypophyseal artery aneurysms when conducted in high-volume centers [49]. Ongoing evolution and advancements in the field of cerebrovascular surgery, including high-resolution preoperative imaging, intraoperative navigation, and microsurgical refinements, will further augment precision in the management of aneurysms in the future. Long-term occlusion rates, re-treatment requirements and neurological outcomes of microsurgical versus endovascular strategies will need to be compared in comparative studies to refine patient selection criteria. While technological advances will continually expand the neurovascular treatment armamentarium, this case illustrates the irreplaceable precision of microsurgical expertise in the handling of complex aneurysms.

## 4. Conclusions

Diagnosis of hypophyseal artery ruptured aneurysm is a challenge, but accurate and timely diagnosis, modern imaging studies, and precise surgical techniques make it possible to control a ruptured hypophyseal artery aneurysm. Although with the advent of endovascular therapies in recent decades, there has been a repertoire of choices for treating aneurysms, the need for microsurgical clipping for complex aneurysms, especially those with wide necks, atypical morphologies and anatomical locations, cannot be discounted.

The demonstration of clipping’s durability and efficacy in this case highlights its continued relevance in the day and age of minimally invasive interventions. Patient-specific, multidisciplinary methods that combine high-tech and clinical features may define future improvement in the management of these aneurysms. Innovations such as those in imaging (e.g., HR-VWI) and artificial intelligence-based rupture risk prediction are yielding the potential to improve risk stratification and treatment decisions. Tools that will help intraoperatively (augmented reality navigation, robotic assistance, etc.) are set to offer further advances in surgical precision and patient experience. New techniques, such as bioresorbable stents and intrasaccular flow disruptors, have the potential to increase the number of minimally invasive treatments available for anatomically challenging aneurysms. But these innovations need to be weighed against the unique advantages of microsurgical approaches, especially when direct visualization and manipulation remain paramount.

This case underscores the imperative for a new paradigm in aneurysm management, one that marries modern technologies with the ever-relevant ideal of neurosurgical excellence. Through continued collaboration between neurosurgeons, interventionists, radiologists, and researchers, we will continue to improve our ability to treat even the most difficult of aneurysms safely and effectively.

## Figures and Tables

**Table 1:** Provides an overview of high-impact studies that explore the outcomes of microsurgical clipping, endovascular techniques, and advanced imaging in the management of cerebral aneurysms. Key findings from these studies are contextualized with relevance to the management of wide-necked and complex aneurysms, including those located in challenging anatomical regions such as the hypophyseal artery. The table serves to illustrate the durability of clipping, the limitations of endovascular approaches in certain scenarios, and the role of advanced imaging modalities in guiding treatment strategies.

Study	Design	Population	Treatment Modalities	Key Findings	Relevance to Current Case
ISAT (2002) [26]	Multicenter RCT	2143 patients with ruptured aneurysms	Microsurgical Clipping vs. Coiling	Coiling was associated with lower 1-year morbidity and mortality compared to clipping, particularly for anterior circulation aneurysms. Long-term follow-up showed higher recurrence rates in coiled aneurysms (20–33%).	Supports the decision for clipping in wide-necked or morphologically complex aneurysms like the hypophyseal aneurysm.
Ferreira et al. (2025) [27]	Systematic Review/Meta-analysis	>8000 patients with aneurysms	Clipping vs. Coiling	Clipping had lower long-term recurrence rates (4.3% vs. 20%) and was preferred for wide-necked aneurysms, despite higher perioperative risks.	Highlights the long-term durability of clipping, particularly for cases like this one with a broad-necked aneurysm.
Lawton et al. (2005) [28]	Retrospective Cohort	1200 patients undergoing clipping	Microsurgical Clipping	High-volume centers showed superior outcomes, with complication rates <5% for complex aneurysms.	Reinforces the importance of experienced surgical teams for cases involving technically challenging aneurysms.
Park (2014) [29]	Surgical Series	1500 aneurysms treated via clipping	Microsurgical Clipping	Emphasized the role of meticulous dissection, temporary clipping, and direct visualization in ensuring durable aneurysm occlusion.	Mirrors the technical strategies employed in the current case, including temporary clipping and precise clip placement.
Hoh et al. (2022) [30]	Retrospective Cohort	800 elderly patients with aneurysms	Clipping vs. Endovascular	Demonstrated that microsurgical clipping is a viable option for elderly patients, with comparable outcomes to endovascular approaches in high-volume centers.	Validates the choice of clipping in this elderly patient, considering the aneurysm’s anatomy and comorbidities.
Chamis et al. (2025) [31]	Prospective Cohort	100 patients with wide-neck aneurysms	Flow Diversion vs. Clipping	Flow diverters showed good efficacy but required prolonged antiplatelet therapy, making them less suitable for elderly or high-risk patients.	Highlights the limitations of flow diversion in this case due to the patient’s advanced age and comorbid conditions.
Larsen et al. (2018) [32]	Systematic Review	10,845 aneurysms	Advanced Imaging	High-resolution vessel wall imaging (HR-VWI) provides insights into aneurysm wall pathology, aiding in rupture risk assessment.	Suggests HR-VWI as a potential tool for assessing rupture risk in similar cases, though not utilized in this case.
Kaiser et al. (2021) [33]	Retrospective Cohort	300 aneurysms	Intraoperative ICG Angiography	ICG angiography confirmed complete exclusion of aneurysms in 95% of cases and identified residual flow in 5%, enabling intraoperative corrections.	Aligns with the use of ICG angiography in this case to confirm clip placement and aneurysm exclusion.

## Data Availability

The data presented in this study are available on request from the corresponding author.
